# Structural empowerment and psychosocial safety climate in healthcare: a systematic integrative review

**DOI:** 10.1108/JHOM-02-2026-0233

**Published:** 2026-10-28

**Authors:** Ljudmila Linnik, Jaana Sepp, Karin Reinhold, Mojtaba Vaismoradi

**Affiliations:** Tallinn Health University of Applied Sciences, Tallinn, Estonia; Tallinn University of Technology, Tallinn, Estonia; Faculty of Nursing and Health Sciences, Nord University, Bodø, Norway; Faculty of Science and Health, Charles Sturt University, Orange, Australia

**Keywords:** Structural empowerment, Psychosocial safety climate, Sensemaking, Integrative review, Nursing management

## Abstract

**Purpose:**

Psychosocial safety climate (PSC) is associated with nurses' well-being, yet the organisational conditions under which shared PSC perceptions form remain insufficiently understood. Drawing on structural empowerment (SE) theory, PSC theory, and organisational sensemaking, this review derives propositions on the SE–PSC association in healthcare organisations.

**Design/methodology/approach:**

A systematic integrative review (PRISMA 2020) of five databases identified 30 studies. The synthesis combined deductive framework analysis with abductive theorising; methodological quality was appraised using Joanna Briggs Institute tools.

**Findings:**

Three propositions emerged. In the resulting framework, leadership and governance are conceptualised as organisational cues in collective sensemaking, resource availability as a signal of management commitment, and role clarity and development opportunities as signals of organisational values. Formal arrangements did not consistently correspond to nurses' experienced authority or access. These patterns support an interpretive account in which leadership coherence, resource provision, and accessible development opportunities may be associated with shared PSC perceptions.

**Research limitations/implications:**

Cross-sectional designs preclude causal inference. Empirical support was stronger for Propositions 1 and 2 than for Proposition 3. Validated PSC instruments remain underused; the propositions require longitudinal and multilevel validation.

**Practical implications:**

Nursing management should align leadership practices, resource allocation, and role clarity as credible signals of organisational commitment to psychological health.

**Social implications:**

The framework offers an evidence-informed basis for policies improving nurses' psychosocial work environments and workforce retention.

**Originality/value:**

The review reconceptualises SE as an interpretive context for PSC formation, offering grounded propositions for empirical testing.

## 1. Introduction

Healthcare organisations operate within intensive psychosocial environments that expose nurses to a wide range of occupational risk factors. International research demonstrates that nurses are confronted with excessive workloads, high emotional demands, insufficient managerial support, and chronic understaffing (Getie *et al.*, 2025). These conditions are associated with impaired psychological well-being, elevated burnout, and increased risks to patient safety and healthcare quality (ILO, 2022; WHO, 2022; Getie *et al.*, 2025). Accordingly, preventing psychosocial risks and protecting nurses' mental health have become strategic priorities in healthcare management.

Within this context, psychosocial safety climate (PSC) has gained prominence as a key organisational construct capturing how institutions signal commitment to employees' psychological health. PSC refers to shared perceptions of the extent to which organisational policies, practices, and procedures prioritise preventing psychosocial risks and protecting psychological well-being (Dollard and Bakker, 2010). Empirical evidence indicates that stronger PSC is associated with lower burnout, improved well-being, and higher work engagement among healthcare workers (Havermans *et al.*, 2017; Fattori *et al.*, 2022). Theoretically, Dollard and McTernan's (2011) multilevel model positions PSC as an organisational-level antecedent of job demands and resources, providing a foundation for employee health and motivation.

Despite its scientific and practical relevance, the organisational conditions associated with shared PSC in healthcare organisations remain insufficiently understood. Research suggests that PSC perceptions vary substantially between organisations and across departments within the same organisation, even where formal policies and value statements are uniform (Klein and Kozlowski, 2000). Organisational climate theory posits that climates such as PSC do not arise solely from formalised policies or managerial communication, but develop through employees' day-to-day experiences and collective interpretation of cues signalling organisational priorities (Morgeson and Hofmann, 1999). However, existing literature offers a limited explanation of why identical messages and structures are associated with a coherent PSC in some units while remaining fragmented in others (McLinton *et al.*, 2023).

This variation in PSC suggests that collective sensemaking processes, by which employees notice, interpret, and stabilise organisational cues over time (Weick, 1995), may be central to understanding the association between structural empowerment (SE) and PSC. In complex, high-responsibility environments, such as healthcare, employees rely on cues perceived as consistent, credible, and enduring when forming shared judgements about organisational priorities (Maitlis and Christianson, 2014). While existing research identifies leadership behaviour as closely associated with PSC (Dollard and Bakker, 2010; Lu *et al.*, 2025), the structural conditions under which leadership messages are perceived as credible and sustainable have received little attention. To address this gap, this review examines whether SE, defined as access to information, resources, support, and opportunities (Kanter, 1977, 1993), may be understood not as a direct antecedent of PSC, but as an organisational interpretive context in which people form shared perceptions of organisational commitment to psychological health. Given the heterogeneity of existing evidence, a theory-building approach is warranted. The objective of this review is therefore to develop a theoretically grounded, proposition-based framework of the SE–PSC association in healthcare organisations, guided by the question: What theoretical propositions can be derived from synthesising SE, PSC, and sensemaking theory to explain this association?

## 2. Methods

### 2.1 Design and epistemological orientation

This study adopts a systematic integrative review design to synthesise methodologically and theoretically heterogeneous international evidence, with a focus on conceptual synthesis rather than effect size estimation. Therefore, quantitative meta-analysis was not considered appropriate (Torraco, 2005). The review protocol was prospectively registered in PROSPERO (Registration No. CRD420251018674).

The review was informed by established integrative review frameworks (Whittemore and Knafl, 2005; Torraco, 2005) and guided by Weick's (1995) sensemaking theory as an analytical lens for translating heterogeneous findings into theoretical propositions. Consistent with this interpretive orientation, the proposed framework is not conceived as a causal model; it maps associational patterns and meaning-making processes characterising the SE–PSC relationship in organisational contexts. The review is reported in accordance with the PRISMA 2020 guidelines (Page *et al.*, 2021; Appendix A). The methodological and conceptual logic underpinning the review is illustrated in Figure 1.

**Figure 1:**
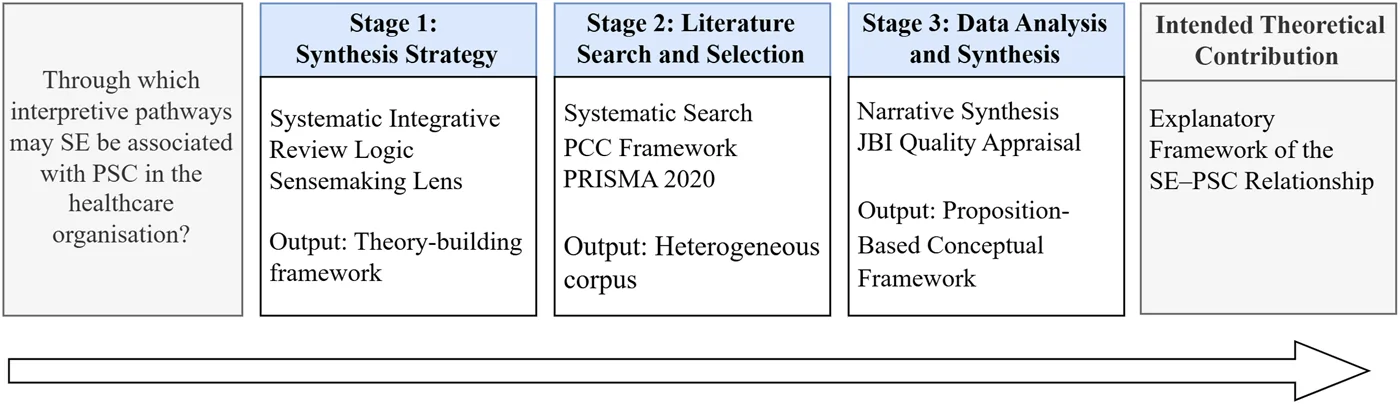
Methodological and conceptual logic of the integrative review

### 2.2 Review scope and eligibility framework (PCC)

Eligibility criteria were defined using the Population–Concept–Context (PCC) framework, which is well-suited to conceptually driven reviews that prioritise constructs and contexts over interventions (Peters *et al.*, 2020). A summary of the eligibility criteria is presented in Table 1.

**Table 1:** Eligibility criteria based on the PCC framework

PCC element	Inclusion criteria	Exclusion criteria
Population (P)	Practising nurses; mixed samples eligible where nurses formed a substantial subgroup	Samples excluding practising nurses
Concept (C1): SE	Organisational-level access to empowering workplace structures operationalised as: (a) validated SE instruments (e.g. CWEQ-II ≥2 of the four core access structures), OR (b) ≥2 core SE dimensions consistent with Kanter (1977, 1993)	SE examined in isolation from PSC or conceptually adjacent climate constructs
Concept (C2): SE–PSC Relationship	Studies examining SE–PSC associations via: (a) validated PSC instruments (PSC-12, PSC-4); (b) ≥2 of 4 PSC core domains; or (c) organisational climate measures relevant to PSC (classified as Conceptual Evidence; §2.6)	Patient safety culture only; PSC without reference to SE
Context (C)	Healthcare organisations; peer-reviewed empirical studies	Non-healthcare settings; non-empirical studies; grey literature

*Population*. Eligible studies focused on practising nurses across clinical, managerial, and advanced practice roles within healthcare settings. Mixed healthcare personnel samples were eligible where nurses constituted a substantial subgroup within a nursing work environment.

*Concept*. SE was required to be conceptualised as nurses' organisational-level access to empowering workplace structures (e.g. information, resources, support, opportunity), consistent with Kanter's structural theory of empowerment (Kanter, 1977, 1993). Quantitative studies typically operationalised SE using the Conditions of Work Effectiveness Questionnaire II (CWEQ-II; ≥2 of the four core access structures; Laschinger *et al.*, 2001), while other validated measures or qualitative approaches examining comparable structural workplace conditions were also eligible.

PSC was required to reflect nurses' shared organisational perceptions of management's commitment to psychological health, consistent with Dollard and Bakker's (2010) theory. Studies were eligible if PSC was measured using validated instruments (PSC-12, PSC-4) or by assessing ≥2 of its four core domains (management commitment, management priority, organisational communication, and participation). Studies employing general organisational climate measures were eligible for inclusion as Conceptual Evidence only, a category of studies that do not directly measure PSC but examine conceptually adjacent constructs (Section 2.6).

*Context*. Eligible contexts were restricted to healthcare organisations, focusing on peer-reviewed empirical studies. Non-empirical publications and grey literature were excluded.

### 2.3 Search methods

A systematic literature search was conducted across five electronic databases: PubMed/MEDLINE, Scopus, Web of Science, CINAHL (via EBSCOhost), and APA PsycINFO (via EBSCOhost). The strategy was developed in collaboration with a research librarian, pilot-tested in PubMed, and adapted for each database to address heterogeneous terminology and inconsistent indexing of SE and PSC. The search was conducted without date or language restrictions; publication-type filters (peer-reviewed journal articles) and, where available, a nursing subject-area filter were applied, consistent with the eligibility criteria (Table 1). English-language eligibility was assessed at full-text screening (Pieper and Puljak, 2021). The final search was completed on 30 September 2025.

The strategy combined concept-based free-text terms, database-specific controlled vocabulary for the population block, where available, and the names of seminal theorists (Kanter, Dollard, Laschinger) and validated instruments (CWEQ-II, PSC-12, PSC-4) as search terms to maintain theoretical alignment. The nursing subject-area filter reflected the population focus of the review; the potential exclusion of relevant studies published in occupational health, psychology, or management journals not indexed under nursing was mitigated through backward and forward citation tracking. The search strategy comprised three search-term blocks: population, SE, and PSC. Within each block, terms were combined with OR; the SE and PSC blocks were then combined using OR, and the resulting set was combined with the population block (nurses or nursing staff) using AND. While the eligibility criteria distinguish two conceptual elements (C1 and C2; Table 1), the search required three term blocks, as the SE–PSC relationship (C2) cannot be searched directly and was instead operationalised through separate SE and PSC term sets. Requiring the SE and PSC concepts to co-occur would have excluded studies addressing either construct in conjunction with relevant climate measures; the broader OR combination was therefore adopted to maximise sensitivity, with conceptual linkage assessed at the screening stage. The search was supplemented by backward and forward citation tracking using Web of Science and Google Scholar; the latter was used for citation tracking only, not as a primary search database (Greenhalgh and Peacock, 2005). Complete database-specific search strategies, including all search terms, limits, and filters, are provided in Appendix B in accordance with the PRISMA extension for reporting literature searches (Rethlefsen *et al.*, 2021).

### 2.4 Screening process

The retrieved records were screened in multiple stages: duplicate removal, title and abstract screening, and full-text assessment based on the predefined eligibility criteria (Table 1). Duplicates were removed automatically in EndNote and verified manually by the first review author (L.L.). Title and abstract screening and full-text assessment were conducted independently by the first two authors; at title and abstract screening, borderline records were advanced to full-text review, and disagreements at either stage were resolved through consensus. Three discrepancies arose at the full-text stage, all concerning whether conceptually adjacent constructs met the PSC-relevance criterion (Trus *et al.*, 2018; Zhao and Yin, 2023; Hirvikallio *et al.*, 2024); these were resolved through joint re-examination and retained as Conceptual Evidence (Section 2.2). Per the pre-registered protocol, the third author (K.R.) was designated as adjudicator, and no third-party adjudication was required. Of the three records identified through citation searching, one full text could not be retrieved; the remaining two were assessed and incorporated into the review (see Section 3.1 and Figure 2).

**Figure 2:**
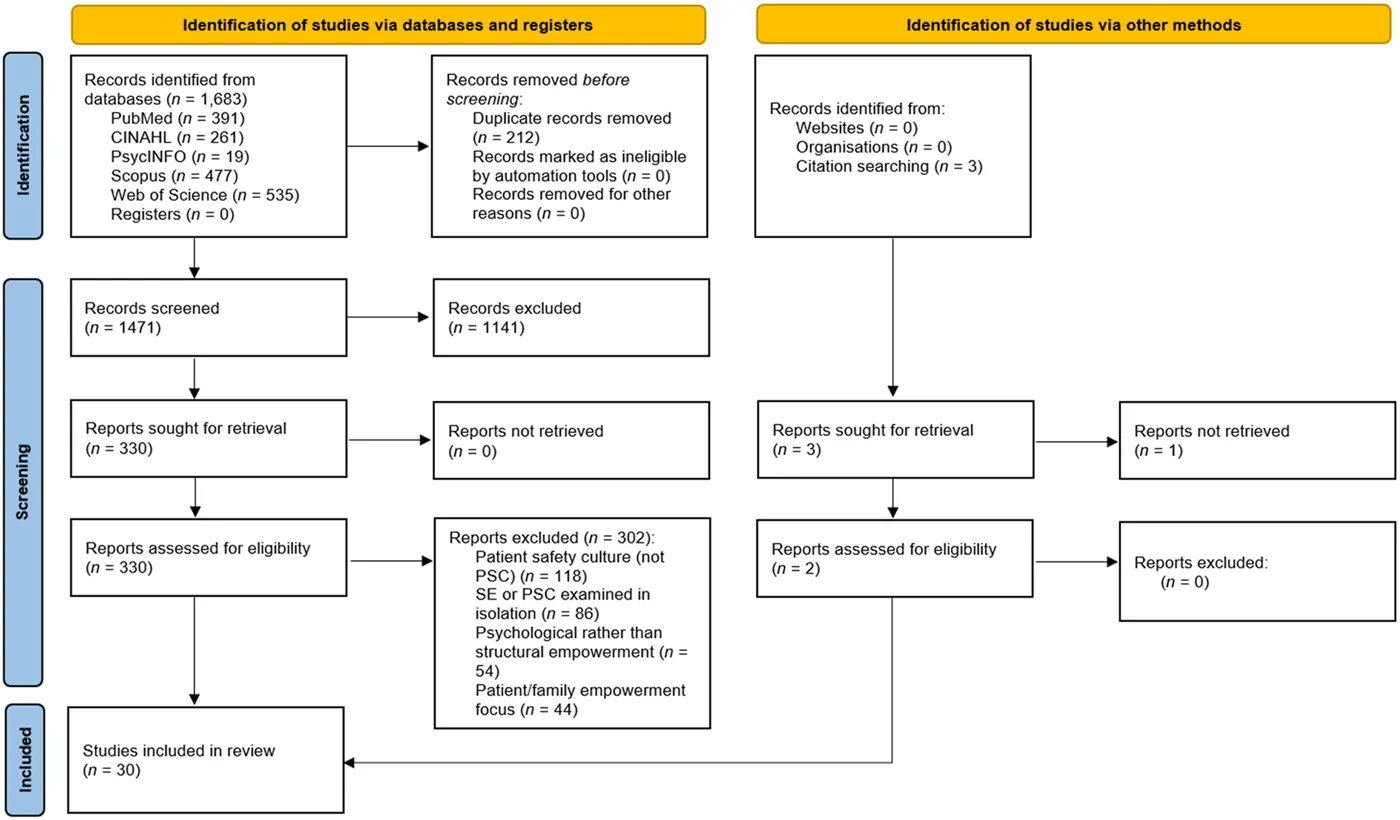
PRISMA 2020 flow diagram

Inter-rater agreement at the full-text screening stage was 99.1% (327 of 330 assessments concordant), corresponding to a Cohen's kappa of κ = 0.94, indicating almost perfect agreement (Landis and Koch, 1977). Since most records were excluded by both authors (302/330), the κ value should be interpreted alongside the percentage agreement, as extreme category prevalence can distort kappa estimates.

### 2.5 Methodological quality appraisal

The methodological quality was appraised independently by two reviewers (L.L. and K.R.) using the Joanna Briggs Institute (JBI) critical appraisal tools appropriate for each study design (Joanna Briggs Institute, 2020; Munn *et al.*, 2020). Although the protocol initially specified both the JBI critical appraisal tools and the mixed methods appraisal tool (MMAT), the JBI tools were applied to all included studies to ensure a consistent appraisal framework across study designs.

Initial independent appraisals demonstrated complete agreement for 27 of the 30 included studies (90%). Discrepancies in the remaining three studies all concerned a single appraisal domain*—*the adequacy with which confounding factors were identified and addressed. Each checklist item was rated as “yes”, “no”, “unclear”, or “not applicable”; “yes” responses were considered criteria met, responses of “no” and “unclear” were treated as criteria not met, and “not applicable” items were excluded from the denominator when calculating overall quality scores.

Since the JBI does not prescribe fixed cut-off scores, pre-specified quality thresholds were applied: studies meeting more than 75% of applicable criteria were classified as high quality, those meeting 50–75% as moderate quality, and those meeting less than 50% as low quality; scores falling exactly at the 75% threshold were classified conservatively as moderate quality. No studies were excluded based on methodological quality. The appraisal was used to characterise the strength of the evidence base and to contextualise the interpretation of the synthesised findings (see Section 3.3). Detailed quality appraisal results are presented in Appendix C.

### 2.6 Data analysis and synthesis

Data extraction was conducted by the first two review authors (L.L. and J.S.) using a standardised form capturing study characteristics, methodological features, and key findings relevant to SE and PSC. Discrepancies were resolved through consensus, with the third (K.R.) and fourth (M.V.) authors providing adjudication where necessary. The synthesis was guided by framework analysis (Gale *et al.*, 2013) but adapted to serve the review's theory-building objective (Torraco, 2005; Whittemore and Knafl, 2005). The initial analytical framework was deductively derived from Kanter's (1977, 1993) theory of SE and Dollard and Bakker's (2010) theory of PSC.

The central methodological challenge was integrating PSC-specific studies (*n* = 6) with conceptually adjacent literature (*n* = 24). We employed a stratified analytical approach in which Core Evidence (*n* = 6) established the model's primary conceptual foundations, while Conceptual Evidence (*n* = 24) served primarily to enrich the interpretive analysis. This structure allowed each proposition to be anchored in at least one PSC-specific study, although much of the interpretive elaboration relied on Conceptual Evidence. The implications of this proportional imbalance for the robustness of the derived propositions are further addressed in Section 5. This synthesis involved four iterative stages through abductive reasoning. First, findings were open-coded to identify meaning units related to SE, organisational conditions, and psychosocial processes. Second, codes were grouped into categories reflecting recurrent patterns. Third, categories were mapped onto the framework. Fourth, where patterns diverged, abductive theorising explored alternative linkages via Weick's (1995) sensemaking theory and constant comparison. Coding and category development were conducted by the first two authors and discussed iteratively, with disagreements resolved through consensus and consultation with the third and fourth authors (K.R.; M.V.) where necessary (see Table 2). The final synthesis, grounded in Core Evidence, also functioned as a sensitivity analysis, assessing the robustness of the propositions to the inclusion of conceptually adjacent literature.

**Table 2:** Illustrative examples of the analytic process from empirical findings to theoretical propositions

Empirical finding	Initial code	Category	Linked proposition
PSC correlated positively with perceived co-worker support; higher baseline PSC at team and individual levels was associated with lower subsequent emotional exhaustion (McLinton *et al.*, 2023)	Management-related climate perceptions associated with peer support and subsequent emotional exhaustion	Leadership as a Central Source of Collective Sensemaking	Proposition 1
Higher PSC was associated with greater job resources and better safety performance (Abdi *et al.*, 2023)	Availability of job resources	Resource Availability as an Interpretive Signal	Proposition 2
Nurses described role clarity and learning opportunities as components of hospital climate (Sein *et al.*, 2021)	Roles and learning as climate components	Role Clarity and Development Opportunities as Organisational Value Signals	Proposition 3

## 3. Results

### 3.1 Study selection

Figure 2 summarises the process of study selection based on PRISMA 2020; Page *et al.* (2021). The initial database search yielded 1,683 records (CINAHL via EBSCOhost, *n* = 261; APA PsycINFO, *n* = 19; PubMed, *n* = 391; Scopus, *n* = 477; Web of Science, *n* = 535). No additional records were identified through registers (*n* = 0). Following importation into EndNote, 212 duplicates were removed (automated deduplication, *n* = 208; manual verification, *n* = 4), resulting in 1,471 records for screening. Two reviewers independently screened all titles and abstracts against the predefined criteria; 1,141 records were excluded, and full texts were sought for the remaining 330 reports, all of which were successfully retrieved and assessed for eligibility. The same two-reviewer independent assessment process was applied at full-text screening. At this stage, 302 reports were excluded based on predefined criteria. The primary reasons for exclusion were: patient safety culture rather than PSC (*n* = 118); SE or PSC examined in isolation (*n* = 86); psychological rather than structural empowerment (*n* = 54); and empowerment of patients or families rather than nurses (*n* = 44).

Forward and backward citation searching of all 28 included studies was conducted in Scopus and Web of Science in September 2025, identifying three additional articles. Of these, one full text could not be retrieved despite direct contact with the corresponding author and interlibrary loan requests, and two met the inclusion criteria. In total, 30 studies were included, consisting of 28 from database search and 2 from citation searching (Figure 2).

### 3.2 Study characteristics

The 30 studies were published between 1996 and 2025 (Table 3). Study designs were predominantly cross-sectional (*n* = 23; 76.7%; including one cross-sectional secondary analysis of longitudinal data (Boamah and Laschinger, 2015)), followed by longitudinal (*n* = 3; 10.0%), qualitative (*n* = 2; 6.7%), mixed-methods (*n* = 1; 3.3%), and cluster randomised controlled trial (*n* = 1; 3.3%). SE was most commonly measured using the Conditions of Work Effectiveness Questionnaire II (CWEQ-II) (*n* = 15; 50.0%). The remaining studies employed alternative validated instruments capturing access to SE.

**Table 3:** Overview of studies included in the integrative review, with proposition assignments and key findings

Author(s), year, country	Study design, sample, analysis, quality rating	Instruments used	P	Key findings
Nursalam *et al.* (2018) ^b^, Indonesia	Cross-sectional; *n* = 134 nurses: inpatient (55), intensive care unit (38), operating room (22), outpatient (19); PLS-SEM; Moderate	CWEQ-II; Job Activities Scale; Organisational Relationship Scale; Psychological Empowerment Scale; MBI; QNWL	P2; P3	SE→PE path coefficient 0.440; PE→burnout −0.371; burnout→QNWL −0.320; SE→QNWL (direct) 0.345; no significant SE→burnout path was retained; indirect SE→PE→burnout→QNWL = 0.052, weaker than the direct SE→QNWL path; total SE effect on QNWL = 0.397
Sein *et al.* (2021), Myanmar	Qualitative; *n* = 65 nurses and managers (17 in-depth interviews, 8 focus groups); content analysis; High	In-depth interview and focus-group guides	P3	Role clarity, learning opportunities, and recognition identified as core SE-enabling climate domains in Myanmar hospitals
Heron and Bruk-Lee (2020), USA	Cross-sectional; *n* = 153 (89.6% registered/licensed practical nurses), various departments; PROCESS macro; Moderate	CWEQ-II; Affective Organizational Commitment Scale; Nursing Workaround Scale; Safety Performance Scale; Nurse Stress Scale	P2	SE was associated with organisational commitment and safety performance. Nurse stress moderated indirect SE–outcome associations: commitment and safety-performance associations were weaker at higher stress, whereas the workaround association was significant only at high stress
Choi and Kim (2019), South Korea	Mixed-methods; cross-sectional survey *n* = 208 nurses; follow-up interviews *n* = 8; SEM; Moderate	CWEQ-II Related: Professional Governance; Autonomy; Job Satisfaction	P1; P3	SE associated with professional autonomy and job satisfaction (β = 0.44* and β = 0.57***); professional governance accounted for part of both associations; workload and hierarchy co-occurred with limited participation
Boamah and Laschinger (2015), Canada	Secondary analysis of second-wave data from a longitudinal study; *n* = 205 nurses; hierarchical regression; High	CWEQ-II (α > 0.70) Psychological Capital Questionnaire (α > 0.70); Utrecht Work Engagement Scale (α > 0.70)	P2; P3	Workplace empowerment and psychological capital were independently associated with work engagement (β = 0.45*** and β = 0.36***; *R*^2^ = 0.38). Opportunity was the highest-rated CWEQ-II subscale (M = 4.01), but this descriptive result was not used as P3 evidence
Lundin *et al.* (2024), Sweden	Cross-sectional; *n* = 312 hospital nursing staff 55% response; generalised estimating equations; High	CWEQ-II (α = 0.91) Climate: Leadership and Management Index (α = 0.96); Individual Care Scale for Nurses (α = 0.90–0.91); Thriving (α = 0.90); Brief Index of Affective Job Satisfaction (α = 0.74); Stress (α = 0.66)	P1; P2; P3	Resources and support were associated with fewer stress symptoms and greater thriving. In adjusted models, opportunities were associated with more frequent individual stress and information with lower unit-level job satisfaction. Leadership showed few well-being associations after SE was included and was inversely associated with staff-rated quality of care at unit level (B = −0.013 and −0.009; both *p* < 0.001)
Kubsch (1996), USA	Qualitative grounded theory; *n* = 36 nurses, 5 health care agencies (1-year observation + interviews); Constant comparative method; Moderate	Participant observation; field notes; informal interviews	P2; P3	Provision diverged from accountability: care plans named independent interventions that job descriptions did not assign to nurses; conflict motivated enactment
Trus *et al.* (2018), Lithuania	Cross-sectional; *n* = 193 (97%) nurse managers, 7 hospitals; Spearman; High	CWEQ-II (α = 0.8); Work Empowerment (α = 0.9)Organizational climate	P1	Proficient/resistant culture and engaged/functional climate associated with higher SE and PE (r < 0.30); less stressful climate independently associated with higher SE
Dwyer *et al.* (2025), USA	Cross-sectional; *n* = 468 US perioperative nurses; multiple regression; High	CWEQ-II (α = 0.88), Psychological Sense of Organisational Membership (α = 0.95); Anticipated Turnover Scale (α = 0.91)	P2; P3	Opportunity was the only CWEQ-II subscale above the scale midpoint (M = 3.62). Belonging predicted lower turnover intent (β = −0.47) more strongly than SE (−0.13); Δ*R*^2^ = 0.278
Çınar and Kutlu (2021), Turkey	Cross-sectional; *n* = 153 surgical nurses, 2 hospitals; multiple regression; Moderate	Structural Empowerment Scale (α = 0.69–0.90); Patient Safety Scale; Empowerment Scale; Employee Safety Culture Scale	P1; P2	SE associated with patient safety culture (β = 0.498***) and employee safety culture (β = 0.330**)
Mok and Au-Yeung (2002), Hong Kong	Cross-sectional; *n* = 331 (50%): 21 top leaders, 55 mid-level managers, 255 staff nurses; multiple regression; Moderate	Modified Litwin and Stringer Organizational Climate Questionnaire (six exploratory factor analysis–derived factors, α = 0.70–0.76); Empowerment scale (α = 0.71–0.81)	P1; P3	Six exploratory factor analysis–derived climate factors explained 44% of variance in empowerment (adjusted *R*^2^ = 0.436***); teamwork (r = 0.51**) and leadership (r = 0.46**) strongest
Laschinger *et al.* (2001), Canada	Cross-sectional; *n* = 404 (72%) nurses, Ontario; SEM; High	CWEQ-II (α = 0.82–0.93), Psychological Empowerment Questionnaire (α = 0.87–0.92); Job Content Questionnaire (α = 0.74–0.85); General Social Support Scale (α = 0.88)	P2	SE was associated with PE (β = 0.85), which was in turn associated with lower job strain (−0.57) and higher work satisfaction (0.79)
Clavelle *et al.* (2013), USA	Cross-sectional; *n* = 95 chief nursing officers, 107 Nurse Practice Council chairs from 344 Magnet orgs (29% response); correlations, regression; Moderate	Index of Professional Nursing Governance (α = 0.959); Nursing Work Index–Revised (α = 0.93)	P1	Shared governance associated with the nursing practice environment (r = 0.416***); control over personnel remained management-dominated
Li *et al.* (2008), Taiwan	Cross-sectional; *n* = 178 (79.9%) nurses, long-term care; multiple regression; Moderate	CWEQ-II (α = 0.90); PE; Work Stress (α = 0.92)	P2	SE inversely associated with work stress (r = −0.44**); the resources subscale was the strongest correlate (r = −0.46***) and was retained in the final stress model (*R*^2^ = 0.32)
Ahmad and Oranye (2010), Malaysia and England	Cross-sectional; *n* = 556 nurses, 2 teaching hospitals; Spearman, regression; Moderate	SE scale (α = 0.86/0.87); Psychological Empowerment scale (α = 0.90); Index of Job Satisfaction; Organizational Commitment scale	P1	Malaysian nurses reported higher SE and organisational commitment, whereas English nurses reported higher job satisfaction. SE–commitment associations were weak in both samples (*ρ* = 0.218 and 0.195); 0.482 refers to affective commitment–job satisfaction, not SE–commitment
Greco *et al.* (2006), Canada	Cross-sectional; *n* = 322 (69%) hospital nurses; path analysis; Moderate	CWEQ-II (subscales α = 0.67–0.95); Leader Empowering Behaviour Scale (α = 0.97); Work life: Areas of Worklife Survey (α = 0.62–0.88); Burnout: MBI-GS EE (α = 0.93)	P1; P3	Leader empowering behaviour→SE (b = 0.71); SE→worklife fit (b = 0.67); worklife fit→burnout/engagement (b = −0.54); no direct leader empowering behaviour–burnout association (*R*^2^ = 0.30)
Yıldız *et al.* (2020), Turkey	Cross-sectional; *n* = 377 (61.6%) nurses, 5 hospitals; path analysis; Moderate	CWEQ-II (α = 0.92); PE (α = 0.93); Occupational Health and Safety Obligations Compliance Scale (α = 0.93); Universal Precautions Compliance Scale (α = 0.86)	P2	SE total effects on occupational health and safety obligations compliance and on universal precautions compliance were β = 0.36*** and β = 0.53***, respectively
Lu *et al.* (2025), China	Cross-sectional; *n* = 676 clinical nurses (7% managers); SEM; Moderate	Head nurse empowerment (α = 0.989); Organizational Climate (α = 0.983); Innovation (α = 0.939); autonomy (α = 0.989)	P1	Head-nurse empowerment was associated with innovative behaviour (total β = 0.635), with organisational climate and professional autonomy represented as intervening variables (indirect β = 0.450; 70.7% of the total association). Cross-sectional design; source tables contain internal inconsistencies
Cho *et al.* (2006), Canada	Cross-sectional; *n* = 226 (58%) newly graduated nurses; SEM; High	CWEQ-II (α = 0.87) Areas of Worklife Survey (α = 0.89); MBI-GS EE (α = 0.91); Affective Commitment Scale (α = 0.79)	P3	SE associated with work-life fit across six domains (b = 0.69) and directly with organisational commitment (b = 0.47***)
Laschinger *et al.* (2009), Canada	Cross-sectional; *n* = 185 (58%) new graduates, *n* = 294 (69%) experienced nurses; SEM; Moderate	CWEQ-II (α = 0.88) Utrecht Work Engagement Scale (α = 0.92); Psychological Work Environment (α = 0.91)	P3	SE associated with work engagement (b = 0.68***); engagement explained the SE effectiveness association in both nurse groups
Hirvikallio *et al.* (2024), Finland	Cross-sectional; *n* = 4,573 (including 366 nurse managers, 8%); mediation; Moderate	Social support (α = 0.85); Psychological Safety (α = 0.8); Commitment (α = 0.7)	P3	Psychological safety associated with organisational commitment (β = 0.46***); social support partially explained the association
Al-Ghwary *et al.* (2024), Jordan	Cross-sectional; *n* = 405 (47%) nurses; correlations and multiple regression; Moderate	CWEQ-II; Work Environment Support Scale (total α = 0.95)	P2; P3	Overall work environment weakly associated with SE (r = 0.200*, standardised β = 0.144*; adjusted *R*^2^ = 9.8%); access to opportunity highest SE subscale (M = 3.38); access to information lowest (M = 3.08)
Zhao and Yin (2023), China	Cross-sectional; *n* = 241 healthcare workers (22% nurses, 80% response); Moderate	Job Control (α = 0.846); Social Support scale (α = 0.922) Safety climate (α = 0.836); Safety performance (α = 0.924)	P2; P3	Job Control associated with hospital safety climate and safety performance (β = 0.463*** and β = 0.167***); social support strengthened specifically the Job Control–safety climate association, which held at both high and low support
Fattori *et al.* (2022), Italy^a^	Cross-sectional (multilevel); *n* = 276 healthcare workers (41% nurses), 17 wards; multilevel models; Moderate	Job resources, social support; PSC-4 (α = 0.84); Copenhagen Burnout Inventory; Effort–Reward Imbalance Questionnaire (α > 0.60); job satisfaction	P1; P2	Ward-level PSC associated with lower job demands and greater job resources; PSC associated with lower burnout (B = −0.28***) and higher job satisfaction (B = 0.69***); PSC × effort and PSC × emotional demands interactions on burnout were non-significant
McLinton *et al.* (2023), Australia^a^	3-wave longitudinal multilevel study; *n* = 380 (nurses, allied health, administration) nested in 63 teams across 3 hospitals; hierarchical linear modelling; High	PSC-12; MBI emotional exhaustion; co-worker support (JCQ 2.0)	P1; P2	Team- and individual-level PSC at T1 were associated with lower emotional exhaustion at T2 (γ = −0.25 and −0.11). Reciprocal exhaustion–co-worker support associations were observed across waves. Individual-level PSC moderated the co-worker support–exhaustion association (interaction γ = −0.05); team-level moderation was not confirmed, and prior outcomes were not controlled
de Wijn and van der Doef (2022), Netherlands^a^	Longitudinal intervention; *n* = 578 (74%) emergency department nurses, 2.5-year follow-up; linear mixed models; High	Participation, resources, workload, PSC-12 (ω = 0.93); Utrecht Work Engagement Scale, 3-item (ω = 0.77–0.75); MBI-HSS (ω = 0.89–0.92); Leiden Quality of Work Questionnaire for Nurses (ω = 0.61–0.79)	P3	Job resources improved, but well-being did not; gains in staffing and autonomy were reported only where actions fitted risks and were well communicated
Boß *et al.* (2025), Germany^a^	Cluster randomised controlled trial; *n* = 347 nurses, 33 hospitals; 150 completed 6-month follow-up (43%); Bayesian estimation; Moderate	Platform intervention (information, resources, support, opportunities) PSC-12; Perceived Stress Scale Patient Health Questionnaire-8	P1	No clear difference in PSC (d = 0.25; 95% CrI −0.32 to 4.44); lower stress (d = −0.33; 95% CrI −4.35 to −0.48); CrIs in raw-score units
Engström *et al.* (2025), Sweden	Cross-sectional; *n* = 409 (47.6%) telephone nurses, national sample; PROCESS; Moderate	CWEQ-II (α = 0.89) Thriving (α = 0.90); Stress (α = 0.84); Job Performance (α = 0.91)	P2; P3	SE associated with job performance (β = 0.32***) and fewer stress symptoms (β = 0.36***); after thriving was entered, the direct stress coefficient fell to β = 0.10 (*p* = 0.108); indirect associations were 0.12 and 0.26
Abdi *et al.* (2023), Iran^a^	Cross-sectional; *n* = 280 (82%), nurses, 13 hospitals; SEM; Moderate	Job resources, autonomy PSC-12 (α = 0.95); Safety Performance (α = 0.90); Job Demands (α = 0.89)	P2	PSC associated with higher job resources and safety performance (β = 0.36*** and β = 0.19**); lower job demands (β = −0.32***)
Bourgoin Boucher *et al.* (2024), Canada^a^	3-wave longitudinal (10 months); *n* = 139 (69 healthcare, 70 education professionals); Mplus; High	Social support, job resources; PSC-4 (α = 0.922); Utrecht Work Engagement Scale, 3-item (α = 0.721); MBI-GS EE (α = 0.917)	P2	PSC at T1 associated with lower psychological demands and higher social support at T2; PSC higher in education than healthcare (d = 0.43*)

Note(s):

^a^ Core Evidence: studies using validated PSC instruments (*n* = 6); unmarked studies constitute Conceptual Evidence (*n* = 24; see Section 2.6). Core Evidence by proposition: P1 (Fattori; McLinton; Boß); P2 (Fattori; Abdi; McLinton; Bourgoin Boucher); P3 (de Wijn and van der Doef). Studies were assigned to the proposition(s) whose constructs they most directly inform. P1 = Leadership and Governance as Organisational Cues in Collective Sensemaking; P2 = Perceived Resource Availability as an Interpretive Signal; P3 = Role Clarity and Development Opportunities as Organisational Value Signals

*Designs are cross-sectional, and analyses are named as reported by the original authors, unless otherwise stated.* Arrows (→) and terms such as “predicted”, “moderated” and “explained” reproduce the analytical language of the original studies and do not represent causal claims of this review; all findings are synthesised as patterns of association. For qualitative studies, Key Findings reports themes as described by the original authors, not measured associations. Quality ratings follow JBI critical appraisal tools: High (>75% of applicable criteria met); Moderate (50–75%); Low (<50%)

α = Cronbach's alpha; ω = McDonald's omega; β = standardised regression or path coefficient; *B* = unstandardised regression coefficient; *b* = unstandardised path coefficient; *r* = Pearson correlation; *ρ* = Spearman correlation; *d* = Cohen's *d*; γ = multilevel model estimate; Δ*R*^2^ = change in explained variance between regression steps; *M* = mean; CrI = Bayesian credible interval (raw-score units); T1, T2 = study waves. CWEQ-II = Conditions for Work Effectiveness Questionnaire II; MBI = Maslach Burnout Inventory (GS = General Survey; HSS = Human Services Survey; EE = Emotional Exhaustion subscale); PE = Psychological Empowerment; PSC = Psychosocial Safety Climate (PSC-4, PSC-12 = 4- and 12-item scales); QNWL = Quality of Nursing Work Life; SE = Structural Empowerment; SEM = structural equation modelling (PLS-SEM = partial least squares)

^b^In Nursalam *et al.* (2018) the *t* values in the source article's Table 3 differ from those in its abstract (table values used here). Two hypothesised paths (SE→burnout; PE→QNWL) were tested but omitted from the final model without reported coefficients

*p < 0.05. **p < 0.01. ***p < 0.001

The studies represented four continents and five geographical regions (see Table 3). Studies were conducted in North America (*n* = 10; 33.3%), Europe (*n* = 7; 23.3%), Asia (*n* = 7; 23.3%), the Middle East (*n* = 4; 13.3%), and Oceania (*n* = 1; 3.3%), with one cross-national study (Ahmad and Oranye, 2010) spanning Malaysia and England counted separately.

Consistent with the analytical framework described in Section 2.6, studies were grouped according to their proximity to PSC measurement: (1) Core Evidence, six studies (20.0%) employing validated PSC instruments (PSC-12, Hall *et al.*, 2010; PSC-4, Dollard, 2019); (2) Conceptual Evidence, twenty-four studies (80.0%) employing broader organisational or safety climate instruments (*n* = 11; 36.7%) or organisational constructs theoretically related to PSC formation (*n* = 13; 43.3%), such as perceived organisational support, psychological safety, and work engagement.

### 3.3 Quality assessment

Based on the JBI appraisal, 10 studies (33.3%) were rated as having high methodological quality and 20 (66.7%) as moderate; no study was rated low. Consistent with the integrative review method (Whittemore and Knafl, 2005), the appraisal was used to characterise the strength of the available evidence rather than to determine inclusion, and no study was excluded on quality grounds. The proportion of studies rated high was descriptively greater in the smaller Core Evidence stratum: three of the six studies measuring PSC directly were rated high (50.0%), compared with seven of the 24 Conceptual Evidence studies (29.2%). All four longitudinal or interventional studies in the review measured PSC directly. Accordingly, the propositions developed in Section 4 are presented as theoretically derived patterns of association that remain open to empirical testing, rather than as established relationships between variables.

## 4. Results of the synthesis: interpretive patterns linking SE and PSC

Consistent with the epistemological stance of this integrative review, the proposed framework is non-causal and interpretive. Many of the included studies were designed and analysed within a variance-modelling tradition, treating SE, organisational climate, and psychological health as distinct variables. Their findings are therefore reported in the original authors' terminology, including terms such as “predicted” and “mediated”. Recasting them in interpretive terms would misrepresent the original analyses. The theoretical interpretations presented below are those of the review authors and are distinguished from the empirical findings throughout.

The synthesis of 30 studies identified three theoretical propositions concerning associations between nurses' experiences of SE and shared perceptions of PSC (see Figure 3). The propositions are not mutually exclusive and are not intended to represent directly observed processes; several studies contributed evidence to more than one. In accordance with integrative review methodology (Whittemore and Knafl, 2005; Torraco, 2005), each proposition is presented in two parts: empirical findings, followed by their theoretical interpretation. This structure allows the reader to trace how each proposition was derived from the evidence, and Table 4 maps the studies contributing to each proposition.

**Figure 3:**
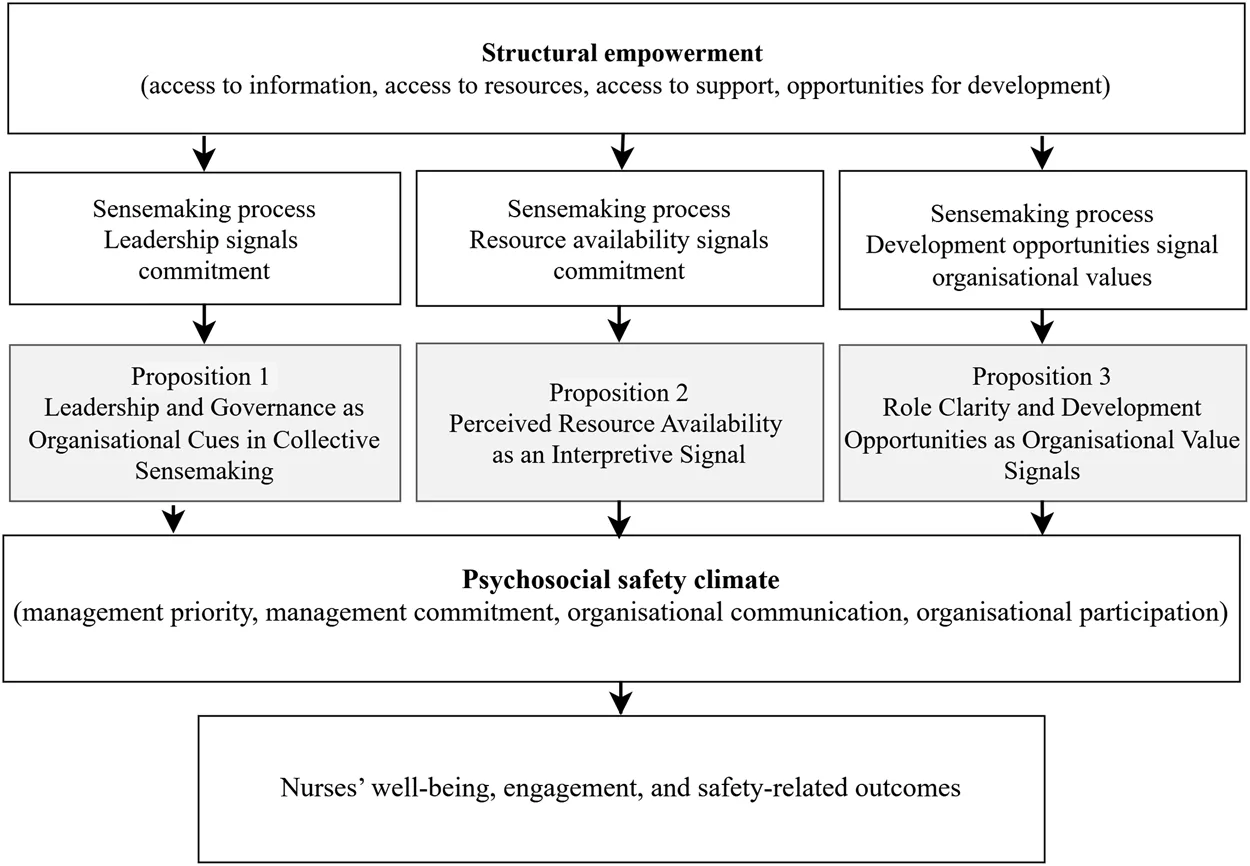
A hypothetical conceptual model linking SE and PSC. Note: The model depicts three parallel interpretive patterns (Propositions 1–3) linking SE components (top box) with PSC (bottom box). Rectangular boxes represent conceptual constructs and core processes. Arrows indicate conceptual associations synthesised from the reviewed literature, not causal claims. Role clarity emerged from the synthesis as an adjacent condition, not a Kanter SE component. The model is based on a synthesis of 30 empirical studies. Source: Authors' own work

**Table 4:** Mapping of empirical evidence to each theoretical proposition

Study	P1	P2	P3
Nursalam *et al.* (2018)	–	SE as a correlate of work-life quality	Psychological empowerment as intermediate state in SE–burnout association
Sein *et al.* (2021)	–	–	Role clarity as an organisational alignment signal
Heron and Bruk-Lee (2020)	–	Stress-contingent SE associations (weaker under high stress)	–
Choi and Kim (2019)	Governance participation as leadership cue; workload as participation barrier	–	Governance accounting for SE–autonomy and SE–satisfaction associations
Boamah and Laschinger (2015)	–	SE and psychological capital as independent engagement correlates	Access to development opportunities as a potential organisational value signal
Lundin *et al.* (2024)	Few independent leadership associations after SE adjustment; inverse care-quality association	Resource access as a stress correlate; information and opportunity contrary to expectation	Opportunity associated with higher individual-level stress
Kubsch (1996)	–	Supportive practice conditions as nursing autonomy context	Role definition–authority mismatch; intra-role conflict
Trus *et al.* (2018)	Weak culture–empowerment associations among nurse managers	–	–
Dwyer *et al.* (2025)	–	SE access as organisational retention signal	Development opportunity access as an empowerment signal
Çınar and Kutlu (2021)	SE as patient and employee safety culture correlate	Structural resources as safety culture correlate	–
Mok and Au-Yeung (2002)	Supportive leadership and teamwork as empowering organisational conditions	–	Training opportunities as an empowerment indicator
Laschinger *et al.* (2001)	–	SE as a correlate of psychological work experience	–
Clavelle *et al.* (2013)	Shared governance–practice environment association; management-dominated control	–	–
Li *et al.* (2008)	–	Resource availability as a work stress correlate	–
Ahmad and Oranye (2010)	Weak SE–commitment associations in two national samples	–	–
Greco *et al.* (2006)	Leader empowering behaviour as SE and work–life fit correlate	–	Person–job fit as intermediate state in SE–burnout association
Yıldız *et al.* (2020)	–	Structural resources as safety behaviour correlate	–
Lu *et al.* (2025)	Head-nurse empowerment as innovation correlate via climate and autonomy	–	–
Cho *et al.* (2006)	–	–	Development opportunity access as work-life fit correlate
Laschinger *et al.* (2009)	–	–	Development opportunity access as effectiveness correlate
Hirvikallio *et al.* (2024)	–	–	Psychological safety as commitment correlate
Al-Ghwary *et al.* (2024)	–	Work environment as modest SE correlate; information lowest-rated subscale	Opportunity as highest-rated SE subscale
Zhao and Yin (2023)	–	Job control as safety climate correlate, amplified by social support	Job control as shared safety-climate correlate; role-autonomy reading
Fattori *et al.* (2022)^a^	Ward-level PSC as burnout correlate	PSC as ward-level job resource and well-being correlate	–
McLinton *et al.* (2023)^a^	PSC at T1 as later emotional exhaustion correlate	Reciprocal support–exhaustion associations; individual-level PSC moderation	–
de Wijn and van der Doef (2022)^a^	–	–	Condition improvements insufficient without interpretive process
Boß *et al.* (2025) ^a^	Imprecise PSC intervention estimate	–	–
Engström *et al.* (2025)	–	Resource access and thriving as correlates of work outcomes	Thriving as intermediate state in SE–stress association
Abdi *et al.* (2023)^a^	–	PSC as resource availability and safety performance correlate	–
Bourgoin Boucher *et al.* (2024)^a^	–	PSC as demand reduction and social support correlate	–

**Note(s):** P1–P3 = Propositions 1–3; “–” indicates no evidence contributed to that proposition. *Entries are interpretive labels summarising each study's contribution; statistical estimates are reported in Table 3*

*Propositions.* P1 = Leadership and Governance as Organisational Cues in Collective Sensemaking; P2 = Perceived Resource Availability as an Interpretive Signal; P3 = Role Clarity and Development Opportunities as Organisational Value Signals

^a^ Core Evidence: studies employing validated PSC instruments (PSC-12 or PSC-4; *n* = 6; see Section 2.6). Each proposition is supported by at least one Core Evidence study

### 4.1 Proposition 1: leadership and governance as organisational cues in collective sensemaking

*Empirical findings.* Twelve studies contributed evidence to Proposition 1 (Table 4). Three are Core Evidence studies employing validated PSC instruments (Fattori *et al.*, 2022; McLinton *et al.*, 2023; Boß *et al.*, 2025); nine are Conceptual Evidence studies. A recurrent pattern across these studies suggests that governance arrangements alone may be insufficient to support well-being: favourable PSC perceptions appear more likely when such arrangements correspond to conditions that nurses experience as structurally empowering.

Leadership, governance, and climate indicators were generally associated with empowerment-related conditions and adjacent organisational or well-being outcomes for nurses. Leader empowering behaviour was associated with higher SE and, via its association with SE, better work–life fit (Greco *et al.*, 2006). Head-nurse empowering behaviour was associated with innovative behaviour, partly via organisational climate and professional autonomy (Lu *et al.*, 2025). SE was associated with stronger patient and employee safety culture (Çınar and Kutlu, 2021). Organisational-climate factors, notably leadership and teamwork, accounted for 44% of the variance in psychological empowerment (Mok and Au-Yeung, 2002). Two studies measured PSC directly, though neither assessed leadership behaviour. Ward-level PSC was associated with lower burnout (B = −0.28; Fattori *et al.*, 2022). Team- and individual-level PSC at Time 1 were associated with lower emotional exhaustion at Time 2 (γ = −0.25 and −0.11; McLinton *et al.*, 2023).

However, this favourable pattern was neither automatic nor universal. In a multilevel Swedish study, leadership ratings were inversely associated with staff-rated quality of care at the unit level (B = −0.013; *p* < 0.001, Model 2), and once access to SE was included, leadership showed few associations with nurses' well-being (Lundin *et al.*, 2024). Heavy workloads and hierarchical culture constrained nurses' participation in governance even where managers were perceived as supportive (Choi and Kim, 2019). In Magnet organisations, shared governance correlated with the nursing practice environment (r = 0.416), yet management remained the dominant controlling group (Clavelle *et al.*, 2013). Among nurse managers, organisational cultures characterised by proficiency and engagement were only weakly associated with higher empowerment (r < 0.30; Trus *et al.*, 2018). SE–commitment associations were similarly weak among both Malaysian and English nurses (*ρ* = 0.218 and 0.195; Ahmad and Oranye, 2010). In the cluster-randomised trial, the standardised PSC effect was small (d = 0.25); the corresponding model estimate was imprecise (95% CrI −0.32 to 4.44 in raw-score units) (Boß *et al.*, 2025).

*Theoretical interpretation.* The pattern of divergence described above is compatible with the possibility that an interpretive process is involved in how SE becomes psychologically meaningful for nurses. Kanter's (1977, 1993) theory identifies access to information — being in the know about decisions and priorities — as the first line of organisational power, with managers as its primary gatekeepers. PSC, in turn, reflects a collective reading of organisational intent rather than formal policy (Dollard and Bakker, 2010).

Weick's (1995) sensemaking framework provides a vocabulary for describing how these two accounts may be related in practice. Nurses extract cues from their environment and collectively label them to stabilise shared understandings of organisational priorities. Access to information (Kanter) is proposed as a structural condition that may allow leadership behaviour to be interpreted as a credible organisational cue. Where managers make information accessible and enable genuine participation, nurses may read this as a signal of management priority and commitment to psychological health. Where information is selectively shared or governance is manager-dominated, the same arrangements may be interpreted as weak signals. Formally declared shared governance may be experienced as incoherent where decision-making authority remains with management (Clavelle *et al.*, 2013; Choi and Kim, 2019). This perspective offers one explanation for why leadership showed few independent associations with well-being once structural access was accounted for Lundin *et al.* (2024). Alternatively, as Lundin *et al.* (2024) themselves suggest, first-line managers who have already established structural access may have fulfilled their primary function. In this review, perceived coherence is proposed as an interpretive condition associated with PSC. It refers to the extent to which nursing staff experience declared organisational values as consistent with everyday managerial practice.

P1.
Favourable PSC perceptions may be more likely to co-occur with leadership and governance practices that visibly enact empowering structures. “Walking the walk” denotes perceived coherence: alignment between stated priorities and nurses' and nurse managers' observable access to information, support, and participative decision-making.

### 4.2 Proposition 2: perceived resource availability as an interpretive signal

*Empirical findings*. Seventeen studies contributed evidence to Proposition 2: four Core Evidence and 13 Conceptual Evidence studies (Table 4). The findings organise around three patterns. First, resource access was associated with lower stress and higher engagement. Resources remained a significant predictor of work stress in Li *et al.*'s (2008) regression model and were the strongest structural correlate of fewer stress symptoms in Lundin *et al.* (2024). SE and psychological capital were independently associated with work engagement (Boamah and Laschinger, 2015), and a composite work-environment model accounted for a modest share of the variance in SE (Al-Ghwary *et al.*, 2024). However, in studies employing mediation models, direct paths from SE to well-being outcomes were often absent or attenuated: Laschinger *et al.* (2001) specified no direct path from SE to job strain; Nursalam *et al.* (2018) retained no direct SE–burnout path, modelling the association through psychological empowerment instead; and only Engström *et al.* (2025) reported the attenuation directly, with the SE–stress association falling from β = 0.36 to β = 0.10 (*p* = 0.108) once thriving was included.

Second, associations between resource availability, safety, and retention varied across contextual conditions. SE was associated with safety compliance and safety culture (Yıldız *et al.*, 2020; Çınar and Kutlu, 2021), and job control was associated with safety climate and performance, more strongly where social support was higher (Zhao and Yin, 2023). SE and workplace belonging were independently associated with lower turnover intention (Dwyer *et al.*, 2025). However, these associations were not uniform. SE's indirect associations with commitment and safety performance were weaker under high nurse-reported stress, and its association with workarounds appeared only under high stress (Heron and Bruk-Lee, 2020). In adjusted models, access to information was inversely related to unit-level job satisfaction, and access to opportunity to more frequent stress (Lundin *et al.*, 2024). Kubsch (1996) documented a provision–practice gap: care plans named independent interventions that job descriptions did not assign to nurses, and the resulting intra-role conflict motivated enactment in some nurses and withdrawal in others.

Third, among Core Evidence studies, PSC was consistently associated with resource allocation and well-being. PSC was associated with greater job resources (β = 0.36) and lower job demands (β = −0.32), which in turn showed associations with emotional exhaustion and safety performance (Abdi *et al.*, 2023), and with lower effort (B = −0.36) and higher rewards (B = 1.16), which accounted for the PSC–burnout association in the authors' mediation model (Fattori *et al.*, 2022). PSC at Time 1 was associated with higher social support and recognition at Time 2, which were subsequently associated with greater work engagement (Bourgoin Boucher *et al.*, 2024). In a longitudinal design, the association between co-worker support and later exhaustion varied with individual-level PSC, though team-level moderation was not confirmed (McLinton *et al.*, 2023).

*Theoretical interpretation.* The empirical divergence in how structural inputs are experienced is compatible with the sensemaking logic outlined under Proposition 1: nurses extract cues from their work environment to construct shared understandings of organisational intent. Access to resources (Kanter) is proposed as a structural condition that may allow management's stated priorities to be interpreted as credible organisational cues. Where managers consistently secure and distribute necessary resources, nurses may read this as a tangible signal of management priority and commitment to psychological health. A pattern compatible with resources carrying interpretive significance is found in Lundin *et al.*'s (2024) anomaly: access to resources was associated with lower stress, whereas access to information and opportunity were associated with lower satisfaction and higher stress — suggesting that not all structural inputs are read identically. Where formal provision exists but high stress or conflicting medical cultures prevent nurses from utilising those resources, the same arrangements may be interpreted as weak signals (Heron and Bruk-Lee, 2020; Kubsch, 1996). In this review, perceived resource availability is proposed as an interpretive condition associated with PSC — the extent to which nursing staff experience formal resource allocation as consistent with everyday managerial commitment to their well-being.

P2.
Favourable PSC perceptions may be more likely to co-occur with observable access to the resources, time, and support necessary for safe nursing practice. Resource availability may be read as an interpretive signal of management commitment, rather than acting solely as a direct buffer against job demands. In this sense, “walking the walk” in the resource domain means consistently enacting adequate provision — not merely declaring it.

### 4.3 Proposition 3: role clarity and development opportunities as organisational value signals

*Empirical findings*. Sixteen studies contributed evidence to Proposition 3: one Core Evidence study and 15 Conceptual Evidence studies (Table 4). The findings organise around three patterns. First, development opportunities and related empowering conditions were generally associated with greater autonomy, stronger intention to stay, and more positive work-related appraisals (Laschinger *et al.*, 2009; Cho *et al.*, 2006; Mok and Au-Yeung, 2002; Dwyer *et al.*, 2025; Hirvikallio *et al.*, 2024). Access to opportunity was the highest-rated SE component among new graduate nurses (Boamah and Laschinger, 2015). In the model tested by Choi and Kim (2019), professional governance accounted for the SE–autonomy (β = 0.05) and SE–job satisfaction (β = 0.04) associations. Zhao and Yin (2023) found that job control was associated with hospital safety climate (β = 0.463); in this review, job control is interpreted as a structural condition that may carry interpretive significance for shared climate perceptions.

Second, the value of these conditions depended on their actual accessibility rather than formal existence alone. In the qualitative phase of their mixed-methods study, Choi and Kim (2019) found that heavy workloads prevented nurses from participating in governance structures even when these formally existed. Lundin *et al.* (2024) found that individual-level access to opportunities was associated with higher stress, while being positively associated with job satisfaction and thriving — suggesting a divergence between formal provision and practical accessibility. In Al-Ghwary *et al.* (2024), the information subscale scored lowest among SE components (M = 3.08 out of 5). Kubsch (1996) observed that role conflict arose where formal role definitions did not match nurses' actual authority in practice. Similarly, Sein *et al.* (2021) found qualitatively that nurses in Myanmar hospitals identified well-defined roles, transparent management processes, and adequate information as core conditions for a supportive organisational climate — indicating that such arrangements may be experienced as supportive only where practical clarity is present.

Third, associations between structural conditions and well-being were frequently accounted for partly by intermediate psychological states, a pattern compatible with an interpretive process. de Wijn and van der Doef (2022) found that the association between objective HR practices and well-being depended on how staff perceived and interpreted those practices. In Greco *et al.*'s (2006) model, SE was associated with better work–life fit, which was in turn associated with lower burnout; in Engström *et al.*'s (2025) model, the indirect association with stress symptoms through thriving was 0.26; and in Nursalam *et al.*'s (2018) model, psychological empowerment accounted for the SE–burnout association, as reported under Proposition 2.

*Theoretical interpretation.* Kanter's (1977, 1993) theory identifies access to opportunity and information as core structural conditions for empowerment. Following the same sensemaking logic, nurses may read clear role expectations and genuine development pathways as signals of management's commitment to their professional growth. The divergence in Lundin *et al.* (2024) and the interpretive dependence found by de Wijn and van der Doef (2022) suggest that formal opportunities may need to be experienced as genuine and accessible in order to carry interpretive weight. In this review, perceived clarity of roles and opportunities is proposed as an interpretive condition associated with PSC — the extent to which nursing staff experience formal development paths and role definitions as consistent with everyday managerial practice.

P3.
Favourable PSC perceptions may be more likely to co-occur with clear role expectations and accessible opportunities for professional development. Role clarity and development opportunities may be read as interpretive signals of management's commitment to employee growth and well-being, rather than functioning solely as individual career benefits. In this sense, “walking the walk” in the opportunity domain means consistently enacting genuine development pathways — not merely formalising them.

## 5. Discussion

This systematic integrative review develops a theoretically grounded, proposition-based framework to theorise how SE is associated with the formation of PSC in healthcare organisations. In contrast to the direct-effects models that have dominated much of the research on nurse well-being, our synthesis of 30 empirical studies yields patterns consistent with the possibility that empowering leadership and access to resources, information, support, and opportunities are collectively read as cues to organisational priorities (Greco *et al.*, 2006; Boamah and Laschinger, 2015; Fattori *et al.*, 2022; McLinton *et al.*, 2023).

### 5.1 Theoretical implications: a proposed model of PSC formation

The central theoretical contribution of this review is the reframing of PSC formation as a process of organisational sensemaking (Weick, 1995). This perspective seeks to reconcile contradictory findings in the extant literature. Although numerous cross-sectional studies report strong positive associations between SE and well-being-related outcomes, such as work engagement and lower burnout (Laschinger *et al.*, 2001; Li *et al.*, 2008; Yıldız *et al.*, 2020), others have found these associations to be context-dependent, weakening under conditions of high stress (Heron and Bruk-Lee, 2020). These inconsistencies suggest that structural conditions alone are insufficient for the formation of PSC. Instead, observed associations depend on how employees collectively interpret the authenticity and credibility of these structures in practice (Clavelle *et al.*, 2013; de Wijn and van der Doef, 2022). The proposed framework is explicitly interpretive and associational in nature and does not advance causal claims. However, it offers theoretically grounded propositions regarding the conditions under which shared PSC perceptions may be formed.

This reframing creates a productive tension with earlier, predominantly direct-effects models of SE (Laschinger *et al.*, 2001), which treated SE as a relatively direct predictor of nursing outcomes. Our research synthesis draws on two complementary theoretical traditions: Kanter's (1977, 1993) structural theory of power, which identifies the organisational conditions enabling meaningful work, and Weick's (1995) sensemaking theory, which explains how employees collectively interpret those conditions to form shared understandings. This shifts the conversation from “more SE = better outcomes” to a process-oriented view, where the key question is: under what conditions is SE associated with a shared belief that the organisation genuinely values psychological health?

Furthermore, our synthesis points to a dual function of PSC within the collective sensemaking process, challenging conceptualisations that treat PSC as a unidirectional outcome of structural conditions. Once formed, PSC is proposed to operate both as a product of collective sensemaking and as an organisational signal associated with subsequent employee perceptions and behaviour. PSC appears to develop in association with empowering structures, wherein leadership practices and the availability of job resources are interpreted as clear organisational cues that employees use to construct shared meanings about psychological health priorities. Viewed through a sensemaking lens, these structural and relational factors are best understood as the interpretive resources from which PSC is collectively formed (cf. Greco *et al.*, 2006; Fattori *et al.*, 2022). This account is further supported by evidence that PSC formation is more readily sustained when leaders' consistent behaviours over time reinforce rather than contradict stated organisational priorities (Clavelle *et al.*, 2013; Boß *et al.*, 2025).

Once collectively established, PSC may itself constitute a shared interpretive framework for evaluating subsequent working conditions. This view is consistent with evidence linking positive climate perceptions to higher empowerment (Mok and Au-Yeung, 2002) and linking PSC with subsequent temporal shifts in social support and workload (McLinton *et al.*, 2023). Therefore, consistent with Weick's (1995) principle that sensemaking is driven by plausibility and ongoing social construction, PSC may operate as a dynamic, collectively negotiated resource whose presence or absence is associated with meaningful differences in the conditions under which nurses make sense of, and respond to, their work environment. We advance this dual function as a theoretical possibility requiring direct empirical examination.

### 5.2 Methodological reflections and directions for future research

The propositions advanced in this systematic review are theoretical and await empirical validation. The primary limitation of the synthesised evidence base is the predominance of cross-sectional studies (*n* = 23; 76.7%), which precludes strong causal inferences. This also reflects a structural gap in the nursing management literature. By articulating theoretically grounded interpretive patterns, our model provides a clear roadmap for filling this gap.

A second limitation concerns the relatively small number of studies employing validated PSC instruments (*n* = 6). While this reflects the nascent state of PSC research in nursing contexts rather than being a limitation of the search strategy, it means that the proposed model draws substantially on conceptually adjacent literature (*n* = 24) to generate its theoretical propositions. We addressed this through a stratified analytical approach, treating PSC-specific studies as the primary evidential basis, while also using the broader SE and organisational climate literature to elaborate the interpretive patterns associated with PSC formation. This distinguished the propositions grounded in PSC-specific evidence from those extrapolated from conceptually adjacent literature. The latter are explicitly framed as theoretical extensions requiring direct empirical validation.

Future research should prioritise three areas. First, longitudinal studies are needed to examine reciprocal SE–PSC associations over time (McLinton *et al.*, 2023; de Wijn and van der Doef, 2022). Second, multilevel intervention studies, such as cluster-randomised controlled trials (Boß *et al.*, 2025), are needed to assess whether changes in specific SE components are associated with the proposed interpretive processes and PSC formation. Third, qualitative and mixed-methods studies are essential to capture the collective sensemaking processes through which structural cues are translated into shared psychological health priorities — opening the interpretive “black box” of PSC formation (Weick, 1995; Maitlis and Christianson, 2014).

### 5.3 Practical implications

For nursing management, our review findings support a strategic shift from symbolic gestures to structural action. Wellness campaigns or policy statements alone are unlikely to sustain favourable PSC perceptions unless they are accompanied by consistently enacted structural commitments. When a discrepancy exists between espoused values and structural reality—chronic understaffing, inconsistent resource access—it may be interpreted by employees as a signal that their psychological health is not a genuine organisational priority (Clavelle *et al.*, 2013; Choi and Kim, 2019).

In practice, this might look like replacing declarations with action: scheduling protected one-on-one meetings rather than declaring open-door policies; redesigning workflows and advocating for adequate staffing rather than praising resilience. Access to resources, support, and development opportunities has been associated with lower stress and better well-being (Li *et al.*, 2008; Lundin *et al.*, 2024). These are not fringe benefits but essential infrastructure for a credible commitment to psychological health.

These implications extend beyond the clinical setting. Nurse turnover and burnout impose substantial costs on healthcare systems globally (Getie *et al.*, 2025). Therefore, investments in SE should be viewed as strategic imperatives, not operational luxuries. Accreditation standards should incorporate measures of how structural resources are actually experienced by frontline staff, not merely how they are declared in policy. A workforce that perceives genuine organisational commitment to its well-being may be better positioned to deliver safe, high-quality care, having the potential to strengthen public trust and inform national workforce retention strategies.

## 6. Conclusion

This systematic integrative review proposes a novel, process-oriented model that theorises how SE is associated with PSC formation, conceptualising this relationship as a process of collective sensemaking. By positioning PSC not merely as an outcome of SE but as a shared interpretive framework in which structural conditions are given meaning collectively, this model advances theoretical understanding beyond linear explanations and provides a coherent framework for future research and evidence-based management practice. The central contribution of this work is the assertion that the protection and promotion of nurses' psychological well-being depend less on organisational rhetoric and more on enacted practices and the consistency and credibility with which these practices are implemented.

## Supplementary Material

Data supplement 1

Data supplement 2

Data supplement 3

## Data Availability

The list of studies included in this systematic integrative review and the data extracted and analysed therefrom are available from the corresponding author upon reasonable request.

## Notes

The supplementary material for this article can be found online.
