# Supplementary material for: Structural empowerment and psychosocial safety climate in healthcare: a systematic integrative review
Source: J Health Organ Manag. 2026 Oct 28;40(9):648–73. doi: 10.1108/JHOM-02-2026-0233 (PMC13711294; doi:10.1108/JHOM-02-2026-0233)
Supplement: Supplementary file 2 [file jhom-02-2026-0233_suppl2.docx]

**APPENDIX B**: **Complete Database Search Strategies**

This appendix provides the complete search strategies for all databases searched in this systematic integrative review, reported in accordance with the PRISMA-S extension for reporting literature searches in systematic reviews (Rethlefsen et al., 2021).

Search period: 25 March – 30 September 2025

Date coverage: Database inception to 30 September 2025

Language restrictions: None applied at the search stage; English-language eligibility was assessed during screening

Publication type filter: Peer-reviewed academic journals (applied in all databases)

Subject area filter: Nursing (applied in multidisciplinary databases where available)

Total records retrieved: 1,683

Databases searched: PubMed/MEDLINE, CINAHL (via EBSCOhost), APA PsycINFO (via EBSCOhost), Scopus, Web of Science (Core Collection)

**Database 1: PubMed (MEDLINE)**

Search fields: Title/Abstract; MeSH Terms

Filters applied: Article type: Journal Article; Subject: Nursing

Records retrieved: n = 391

Search string:

(("structural empowerment"[Title/Abstract] OR "Kanter"[Title/Abstract] OR "CWEQ"[Title/Abstract] OR "Laschinger"[Title/Abstract]) OR ("psychosocial safety climate"[Title/Abstract] OR "PSC-12"[Title/Abstract] OR "PSC-4"[Title/Abstract] OR "Dollard"[Title/Abstract])) AND (nurs*[Title/Abstract] OR "registered nurse"[Title/Abstract] OR "Nurses"[MeSH Terms] OR "Health Personnel"[MeSH Terms])

**Database 2: CINAHL (via EBSCOhost)**

Search fields: Text Word (TX) for concept terms; CINAHL Headings (MH) and Text Word (TX) for population terms

Limiters applied: Publication Type: Academic Journal; Subject: Nursing

Records retrieved: n = 261

Search string:

(TX “structural empowerment” OR TX “Kanter” OR TX “CWEQ” OR TX “Laschinger” OR TX “psychosocial safety climate” OR TX “PSC-12” OR TX “PSC-4” OR TX “Dollard”) AND (TX nurs* OR TX “registered nurse” OR (MH “Nurses+”) OR (MH “Health Personnel+”))

**Database 3: APA PsycINFO (via EBSCOhost)**

Search fields: Text Word (TX)

Limiters applied: Publication Type: Academic Journal; Classification: Nursing (3370)

Records retrieved: n = 19

Search string:

(TX "structural empowerment" OR TX "Kanter" OR TX "CWEQ" OR TX "Laschinger" OR TX "psychosocial safety climate" OR TX "PSC-12" OR TX "PSC-4" OR TX "Dollard") AND (TX nurs* OR TX "registered nurse")

**Database 4: Scopus**

Search fields: Title, Abstract, Author Keywords

Limits applied: Document Type: Article; Subject Area: Nursing

Records retrieved: n = 477

Search string:

TITLE-ABS-KEY("structural empowerment" OR "Kanter" OR "CWEQ" OR "Laschinger" OR "psychosocial safety climate" OR "PSC-12" OR "PSC-4" OR "Dollard") AND TITLE-ABS-KEY(nurs* OR "registered nurse" OR nursing OR "healthcare worker")

**Database 5: Web of Science**

Search fields: Topic (TS: Title, Abstract, Author Keywords, Keywords Plus)

Refinements applied: Document Type: Article; Web of Science Categories: Nursing

Records retrieved: n = 535

Search string:

TS=(“structural empowerment” OR “Kanter” OR “CWEQ” OR “Laschinger” OR “psychosocial safety climate” OR “PSC-12” OR “PSC-4” OR “Dollard”) AND TS=(nurs* OR “registered nurse” OR nursing OR “healthcare worker”)

**Citation Tracking:**

Backward citation tracking (screening reference lists of included studies) and forward citation tracking (identifying studies citing included studies) were undertaken to identify theoretically central studies not retrieved through database searches alone.

**Reference:**

Rethlefsen, M. L., Kirtley, S., Waffenschmidt, S., Ayala, A. P., Moher, D., Page, M. J., & Koffel, J. B. (2021). PRISMA-S: an extension to the PRISMA statement for reporting literature searches in systematic reviews. *Systematic reviews*, *10*(1), 39. <https://doi.org/10.1186/s13643-020-01542-z>
