# Supplementary material for: Structural empowerment and psychosocial safety climate in healthcare: a systematic integrative review
Source: J Health Organ Manag. 2026 Oct 28;40(9):648–73. doi: 10.1108/JHOM-02-2026-0233 (PMC13711294; doi:10.1108/JHOM-02-2026-0233)
Supplement: Supplementary file 3 [file jhom-02-2026-0233_suppl3.docx]

**APPENDIX C** Quality Assessment of Included Studies Using Joanna Briggs Institute (JBI) Critical Appraisal Tools (2020)

| **Author (Year)** | **Study Design** | **JBI Score** | **Quality Rating** | **Key Methodological Features** |
| --- | --- | --- | --- | --- |
| Nursalam et al. (2018) | Cross-sectional | 5/8 | Moderate | S: Validated instruments; PLS-SEM.  L: Cross-sectional; single hospital; reliability not reported; weak factor loadings |
| Sein et al. (2021) | Qualitative | 8/10 | High | S: First qualitative study of OC in Myanmar nursing; all tiers of RNs; rich verbatim quotes; dual ethics approval; member checking and triangulation.  L: No reflexivity statement; single-country context; participants' reluctance to voice negatives. |
| Heron and Bruk-Lee (2020) | Cross-sectional | 6/8 | Moderate | S: All instruments validated with reliability reported (α=0.83–0.97); robust analysis (PROCESS, bootstrapping, VIF, post hoc power >0.80); clear inclusion criteria and detailed sample description.  L: Cross-sectional; confounding factors not identified or controlled; self-report /common-method variance; predominantly female, Caucasian, US sample limits generalisability. |
| Choi and Kim (2019) | Sequential explanatory mixed-methods | Quantitative component: 5/8; qualitative component: 7/10 | Moderate | S: Adequate quantitative sample; power analysis; validated instruments; path analysis; qualitative interviews used to explain quantitative findings.  L: Convenience sampling; response rate not reported; small qualitative sample; limited reflexivity and credibility procedures; some low subscale reliability |
| Boamah and Laschinger (2015) | Cross-sectional secondary analysis of longitudinal data | 7/8 | High | S: Registry-based census sampling; validated instruments with good reliability (CWEQ-II α=0.83, PsyCap α=0.88, UWES α=0.85); hierarchical regression.  L: Cross-sectional secondary analysis (authors note it precludes causal inference); 60% response rate may limit generalisability; confounders not controlled in the model. |
| Lundin et al. (2024) | Cross-sectional | 7/8 | High | S: Large, randomised sample (n=312); GEE for clustering; high reliability.  L: Cross-sectional; 55% response rate. |
| Kubsch (1996) | Qualitative | 7/10 | Moderate | S: Methodological congruity; data triangulation; constant comparative method.  L: Reflexivity and researcher positioning not addressed; no explicit mention of ethical approval. |
| Trus et al. (2018) | Cross-sectional | 7/8 | High | S: Use of internationally validated instruments (CWEQ-II, OSC); exceptionally high response rate (97%); detailed description of setting and participants; pilot study conducted; confounding factors identified and measured.  L: Lack of multivariate statistical analysis to control for confounders; cross-sectional design limits causal inferences; significant time lag between data collection (2012) and publication (2019). |
| Dwyer et al. (2025) | Cross-sectional | 7/8 | High | S: Use of validated instruments (PSOM, CWEQ-II, ATS) with high reliability; detailed description of participants and setting; comprehensive ethical approval; identification and statistical control of confounding factors via hierarchical regression.  L: Reliance on subjective self-report for the primary outcome rather than objective clinical data; cross-sectional design limits causal inferences; potential self-selection bias from online convenience sampling. |
| Çınar and Kutlu (2021) | Cross-sectional | 6/8 | Moderate | S: Use of validated instruments; high model explanatory power (R²=0.668 for patient safety culture).  L: Convenience sampling in two hospitals; no statistical control for demographic confounders (age, experience) in regression; reliance on self-report. |
| Mok and Au-Yeung (2002) | Cross-sectional | 5/8 | Moderate | S: Validated instruments with factor analysis; clear setting description; formal ethical approval.  L: Subjective self-report; no statistical control for confounders; 50.3% response rate; single-center study. |
| Laschinger et al. (2001) | Cross-sectional | 7/8 | High | S: Random sampling; high reliability; 72% response rate; SEM.  L: No statistical control for confounders. |
| Clavelle et al. (2013) | Cross-sectional | 6/8 | Moderate | S: Use of validated instruments (IPNG, NWI-R) with high reliability; clear description of Magnet organizations and participant demographics; ethical approval reported.  L: Low response rate (27.6%); reliance on subjective self-reported perceptions for outcomes; limited statistical control for confounding variables in regression; generalizability limited to Magnet organizations. |
| Li et al. (2008) | Cross-sectional | 6/8 | Moderate | S: Validated instruments; 79.9% response rate; clear ethical reporting.  L: Subjective self-report; limited confounder control; limited generalizability. |
| Ahmad and Oranye (2010) | Cross-sectional | 6/8 | Moderate | S: Simple random sampling; cross-cultural design; validated instruments.  L: No statistical control for confounders. |
| Greco et al. (2006) | Cross-sectional | 6/8 | Moderate | S: Random sampling; 69% response rate; validated instruments; SEM analysis. L: Subjective self-report. |
| Yıldız et al. (2020) | Cross-sectional | 6/8 | Moderate | S: Reliable instruments (α=0.86-0.93); path analysis; multi-hospital.  L: Cross-sectional; demographics incomplete. |
| Lu et al. (2025) | Cross-sectional | 6/8 | Moderate | S: Large sample (n=676); very high reliability (α>0.93); SEM.  L: Cross-sectional; convenience sampling. |
| Cho et al. (2006) | Cross-sectional | 7/8 | High | S: Clear theoretical model; validated instruments; 58% response rate; SEM.  L: No statistical control for confounders; cross-sectional design. |
| Laschinger et al. (2009) | Cross-sectional | 6/8 | Moderate | S: Large sample with two cohorts; validated instruments; SEM.  L: Cross-sectional; secondary analysis. |
| Hirvikallio et al. (2024) | Cross-sectional | 6/8 | Moderate | S: Very large sample (n=4,573); mediation analysis.  L: Subjective self-report no statistical control for confounders. |
| AL-Ghwary et al. (2024) | Cross-sectional | 6/8 | Moderate | S: Large sample; reliable instruments (α=0.80-0.95); 47% response rate.  L: Convenience sampling; cross-sectional. |
| Zhao and Yin (2023) | Cross-sectional | 6/8 | Moderate | S: 80% response rate; high reliability (α>0.84); moderated mediation.  L: Subjective self-report for outcomes; limited statistical control for confounding factors; cross-sectional design; convenience sampling. |
| Fattori et al. (2022) | Cross-sectional | 6/8 | Moderate | S: Multilevel analysis for clustering; high response rate.  L: Subjective self-report for outcomes; limited statistical control for confounders; cross-sectional design; single-center study. |
| McLinton et al. (2023) | Longitudinal | 10/11 | High | S: Longitudinal multilevel design; HLM; diverse healthcare staff.  L: Reliability not reported; attrition not addressed. |
| de Wijn and van der Doef (2022) | Longitudinal (3-wave, 2.5-year) intervention study; realist evaluation | 9/11 | High | S: 2.5-year longitudinal design; multilevel modeling; multi-site study.  L: Not free of outcome at baseline; attrition at unit level. |
| Boß et al. (2025) | Pragmatic parallel cluster RCT with embedded qualitative process evaluation | 9/13 | Moderate | S: Independent stratified cluster randomisation; allocation concealment; preregistration; validated measures; ITT Bayesian multilevel analysis accounting for clustering and missing data; sensitivity analysis.  L: The achieved sample was below the prespecified target; only 43% completed the six-month follow-up (approximately 57% attrition); intervention uptake was low; participants, providers, and self-reported outcome assessment were not blinded. |
| Engström et al. (2025) | Cross-sectional | 6/8 | Moderate | S: High reliability (α>0.84); mediation analysis (PROCESS macro).  L: Cross-sectional; 47% response rate. |
| Abdi et al. (2023) | Cross-sectional | 6/8 | Moderate | S: Large sample (n=280); 82% response rate; high reliability (α>0.89); SEM.  L: Subjective self-report for all outcomes; no statistical control for identified demographic confounders; cross-sectional design; convenience sampling. |
| Bourgoin Boucher et al. (2024) | Longitudinal | 10/11 | High | S: Longitudinal 3-wave design; validated instruments; demonstrates temporal precedence.  L: Small sample (n=139); low reliability (UWES-3: α=0.721). |

*Note.* JBI = Joanna Briggs Institute; S = Strengths; L = Limitations. Quality ratings were defined as High (more than 75% of applicable criteria met), Moderate (50–75%), and Low (less than 50%); scores falling exactly at the 75% threshold were classified conservatively as moderate quality. Different JBI critical appraisal tools were used based on study design: analytical cross-sectional studies (8 items), qualitative research (10 items), cohort studies (11 items), and randomised controlled trials (13 items). For the mixed-methods study, the quantitative and qualitative components were appraised separately using the corresponding tools, and the lower of the two ratings was applied. α = Cronbach's alpha; ω = McDonald's omega; PLS-SEM = Partial Least Squares Structural Equation Modelling; SEM = Structural Equation Modelling; GEE = Generalized Estimating Equations; HLM = Hierarchical Linear Modelling; LMM = Linear Mixed Models; RCT = Randomised Controlled Trial.
